# Systemic Human Neutrophil Lipocalin Associates with Severe Acute Kidney Injury in SARS-CoV-2 Pneumonia

**DOI:** 10.3390/jcm10184144

**Published:** 2021-09-14

**Authors:** Sara Bülow Anderberg, Miklos Lipcsey, Michael Hultström, Ann-Katrin Eriksson, Per Venge, Robert Frithiof, on behalf of the Uppsala Intensive Care COVID-19 Research Group

**Affiliations:** 1 Department of Surgical Sciences, Anesthesiology and Intensive Care, Uppsala University, SE-751 85 Uppsala, Sweden; Miklos.Lipcsey@surgsci.uu.se (M.L.); michael.hultstrom@mcb.uu.se (M.H.); robert.frithiof@surgsci.uu.se (R.F.); 2 Hedenstierna Laboratory, Anesthesiology and Intensive Care, Department of Surgical Sciences, Uppsala University, SE-751 85 Uppsala, Sweden; 3 Department of Medical Cell Biology, Integrative Physiology, Uppsala University, SE-751 23 Uppsala, Sweden; 4 Diagnostics Development a P&M Venge Company, SE-753 12 Uppsala, Sweden; ann-katrin@diagnosticsdevelopment.com (A.-K.E.); per.venge@medsci.uu.se (P.V.); 5 Department of Medical Sciences, Clinical Chemistry, Uppsala University, SE-751 85 Uppsala, Sweden

**Keywords:** COVID-19, intensive care, neutrophils, HNL, acute kidney injury

## Abstract

Neutrophils have been suggested mediators of organ dysfunction in COVID-19. The current study investigated if systemic neutrophil activity, estimated by human neutrophil lipocalin (HNL) concentration in peripheral blood, is associated with acute kidney injury (AKI) development. A total of 103 adult patients admitted to intensive care, with PCR-confirmed SARS-CoV-2 infection, were prospectively included (Clinical Trials ID: NCT04316884). HNL was analyzed in plasma (P-HNL Dimer) and in whole blood (B-HNL). The latter after ex vivo activation with N-formyl-methionine-leucine-phenylalanine. All patients developed respiratory dysfunction and 62 (60%) were treated with invasive ventilation. Sixty-seven patients (65%) developed AKI, 18 (17%) progressed to AKI stage 3, and 14 (14%) were treated with continuous renal replacement therapy (CRRT). P-HNL Dimer was higher in patients with invasive ventilation, vasopressors, AKI, AKI stage 3, dialysis, and 30-day mortality (*p* < 0.001–0.046). B-HNL performed similarly with the exception of mild AKI and mortality (*p* < 0.001–0.004). The cohort was dichotomized by ROC estimated cutoff concentrations of 13.2 µg/L and 190 µg/L for P-HNL Dimer and B-HNL respectively. Increased cumulative risks for AKI, AKI stage 3, and death were observed if above the P-HNL cutoff and for AKI stage 3 if above the B-HNL cutoff. The relative risk of developing AKI stage 3 was nine and 39 times greater if above the cutoffs in plasma and whole blood, respectively, for CRRT eight times greater for both. In conclusion, systemically elevated neutrophil lipocalin, interpreted as increased neutrophil activity, was shown to be associated with an increased risk of severe AKI, renal replacement therapy, and mortality in COVID-19 patients with respiratory failure.

## 1. Introduction

Patients suffering from severe acute respiratory syndrome coronavirus 2 (SARS-CoV-2) pneumonia are treated in intensive care in 5–9% of cases [1]. The incidence of acute kidney injury (AKI) is currently estimated to 50% in these patients. ICU mortality initially reported above 50% has since fallen [2,3,4].

Immunomodulatory treatments, such as dexamethasone and tocilizumab, are so far the only interventions that reduce mortality in COVID-19 patients suffering from respiratory insufficiency [5,6,7]. This suggests a dysregulated inflammatory process causing collateral damage to pulmonary and extra-pulmonary tissues. Neutrophils account for 50–70% of circulating leukocytes and are involved in both innate and adaptive immune responses [8]. Quiescent circulating neutrophils become primed and finally activated as they encounter chemokines, cytokines, and pathogen-associated molecular patterns (PAMPs). In their activated state, neutrophils eradicate invading pathogens via exocytosis of degrading enzymes, production of reactive oxygen species (ROS), phagocytosis, and the release of neutrophil extracellular traps (NETs) additionally they perpetuate inflammation by cytokine release [9]. These abilities have the potential to cause direct injury to host tissues and propagate inflammation due to the generation of damage-associated molecular patterns (DAMPs) [10,11]. Emerging evidence suggests that neutrophils may be implicated in critical COVID-19 [12]. Increased neutrophil counts, neutrophil-to-lymphocyte ratios (NLRs), NET concentrations, and an elevated tendency to expel NETs have all been associated with greater disease severity [13,14,15,16]. Neutrophil pulmonary infiltration and NETs have been observed post-mortem in patients who have succumbed to the disease [17].

Human neutrophil lipocalin (HNL), also called neutrophil gelatinase-associated lipocalin (NGAL), is a glycoprotein released by epithelial cells and activated neutrophils [18]. Unprimed neutrophils react less to PAMPs such as the bacterial peptide N-formyl-methionine-leucine-phenylalanine (fMLP), while primed neutrophils react more strongly [19]. In the current study, we measured the neutrophil-specific dimeric HNL (P-HNL Dimer) in plasma as an estimate of systemic neutrophil activity [20,21]. Furthermore, we analyzed the release of HNL from fresh whole blood (B-HNL) after ex vivo activation with fMLP, mainly reflecting neutrophil priming, in addition to neutrophil activity. Neutrophils have previously been implicated in other forms of renal dysfunction in the intensive care setting, such as septic and ischemic AKI [22,23]. We therefore hypothesized that an increase in HNL, interpreted as neutrophil priming and activity, in peripheral plasma and blood associates with acute kidney injury and the risk of death in a cohort of patients with COVID-19 treated under intensive care.

## 2. Materials and Method

This study was approved by the National Ethical Review Agency (EPM, Box 2110, SE-750 02 Uppsala, Sweden) (No. 2020-01623), and the protocol of the study was registered (Clinical Trials ID: NCT04316884). The Declaration of Helsinki and its subsequent revisions were followed, and the STROBE guidelines were adhered to. This prospective observational study was performed at the general intensive care unit (ICU) at Uppsala University Hospital, Uppsala, Sweden—a tertiary hospital.

### 2.1. Data Collection

Clinical data were collected from electronic medical records. Simplified Acute Physiology Score 3 (SAPS3), Sequential Organ Failure Assessment (SOFA), respiratory support, vasopressor, and continuous renal replacement treatment (CRRT), and 30-day mortality, were registered [24,25]. If the SOFA score at admission was missing, the next registered score was used. Respiratory failure was estimated by invasive ventilation treatment, as well as the ratio of arterial partial oxygen pressure (mmHg) and inspired oxygen fraction (PaO_2_/FiO_2_). AKI severity was staged solely according to the Kidney Disease: Improving Global Outcome (KDIGO) creatinine criteria and renal replacement requirement [26]. Thrombosis was defined as clinically significant thromboembolic events, including pulmonary embolism, deep vein thrombosis, arterial thrombosis, and cerebral infarction. Mortality was registered as within 30 days from ICU admission. Free days were calculated as 30 days minus days of relevant treatment multiplied by 30-day mortality, where mortality equaled 0 and survival equaled 1. Time to events were days from symptom debut.

Plasma was collected in EDTA vials at regular intervals during the intensive care period. The samples were centrifuged, separated, and stored at −80 °C. Plasma HNL analysis was performed in samples collected adjacent to ICU admission (*n* = 102) and on ICU day three (*n* = 65). Whole blood assays of HNL were performed in 67 patients, where immediate handling of blood samples was practically feasible.

Routine chemistry, including white blood cell count (WBC), inflammatory markers such as C-reactive protein (CRP), procalcitonin (PCT), and ferritin, and plasma creatinine, were performed in the hospital central laboratory. Leukocyte, neutrophil, and lymphocyte count samples were collected in K2-EDTA vacutainer tubes (354664, Becton Dickinson, Franklin Lakes, NJ, USA). The samples were then analyzed using a Sysmex XN™ instrument (Sysmex, Kobe, Japan). CRP, PCT, ferritin, and creatinine were analyzed on an Architect ci16200 (Abbott Laboratories, Abbott Park, IL, USA). The values used in the tables represent the day of ICU admission and the highest registered value during intensive care, including the day of admission. In case of multiple analyses within 24 h of admission, the average value was used. If only one value was registered during ICU treatment, this was automatically regarded as the highest value. NLR was calculated at admission and at the observed peak neutrophil count.

### 2.2. Human Neutrophil Lipocalin Assays

Whole blood analysis was performed in lithium heparin anticoagulated blood. Neutrophils were activated using fMLP within 2 h of sampling. Blood was incubated for 20 min at 37 °C with 5 × 10^−8^ mol/L fMLP and the activation process was ended by ice-cold EDTA. The samples were then centrifuged and the plasma collected. From every whole blood sample, two aliquots were subjected to separate activation and HNL assay.

A non-activated aliquot of whole blood was used as a negative control [27]. A total of 197 whole blood HNL analyses were performed, ranging between two and 11 consecutive analysis per patient dependent on their length of ICU stay. Circulating levels of dimeric HNL were measured in EDTA-plasma (P-HNL Dimer) without prior activation. ELISA technique was applied to estimate the HNL concentrations. The antibodies used in the whole blood immunoassay were monoclonal (mab) 765 combined with polyclonal antibodies (pab), detecting both dimeric and monomeric HNL [28]. In the plasma immunoassay, monoclonal antibody 763 combined with 765 was used with specific preference for dimeric HNL.

### 2.3. Cytokine Assay

Citrated plasma samples were analyzed at admission for interleukin-2 (IL-2), IL-6, IL-8, IL-10, IL-17A, tumor necrosis factor α (TNF α), IL-1 receptor antagonist (IL-1ra), interferon gamma-induced protein 10 (IP-10), granulocyte colony stimulating factor (G-CSF), monocyte chemoattractant protein-1 (MCP-1), and interferon (IFN) γ with Bio-plex assays using a Luminex MagPix instrument (Bio-Rad Laboratories AB, Sundbyberg, Sweden).

### 2.4. Statistical Analysis

Variables are presented as mean (SD), median (IQR), or number of observations (percent of total number of observations), as appropriate. Logarithmic transformation was used to normalize the variables. Independent *t*-tests or one-way ANOVAs were applied to compare group means. Post-hoc analyses were Bonferroni-adjusted. Repeated tests were assessed using repeated measures ANOVAs. Due to the small number (*n*) of patients treated for more than three weeks in the ICU, a repeated measures ANOVA was not performed past sample four. Linear regression was used to analyze the relationships between continuous variables when logarithmically transformed. Receiver operator curves in conjunction with Youden’s J statistic were calculated to find suitable cutoff values for HNL. Logistic regression was used to analyze dichotomous outcomes. Correlations were calculated using Spearman’s rank order correlation tests. A two-sided *p* < 0.05 was considered significant. All statistical analyses were performed using RStudio version 1.3.1093 and SPSS version 26. Due to the exploratory nature of this study, we did not adjust for multiple statistical testing.

## 3. Results

### 3.1. Patient Demographics, Routine Chemistry, and Organ Support Requirement

All adult patients admitted to the ICU due to respiratory and/or circulatory insufficiency and suspected SARS-CoV-2 infection (*n* = 149) were screened for inclusion between 13 March 2020 and 29 June 2020. Informed consent was obtained from the patient or next of kin if the patient was unable (*n* = 120). Patients were later excluded in the case of chronic immunosuppression, in the event of PCR screening that could not confirm the infection, organ failure due to causes other than SARS-CoV-2, or immediate discharge. In addition, patients had to have plasma samples in the PRONMED Biobank (BbA-827-2018-009) to participate in the present study (*n* = 103) (Figure S1).

Patients were admitted to the ICU a median of 10 days after initial symptoms. The majority of patients were male. Commonly observed comorbidities were hypertension, pulmonary disease, and diabetes mellitus. Invasive ventilation was used in 62 (60%, *n* = 103) patients. AKI was observed in 67 (65.0%) patients. Sixteen (16%) patients reached AKI stage 2, 18 (17%) patients progressed to stage 3, and continuous renal replacement therapy (CRRT) was used in 14 (14%, *n* = 101) patients. Thirty-day mortality was 19% (Table 1).

The cohort demonstrated elevated inflammatory markers such as CRP and ferritin at admission and during intensive care (Table 2). Normal to increased leukocyte counts were generally observed. PCT had a median of 0.46 (0.93) µg/L at admission. IL-6 and IL-8 reached moderate levels in plasma at admission with concentrations of 30.0 (48.7) and 15.1 (14.1) ρg/mL, respectively.

There were no major differences observed in the baseline demography and clinical char acteristics between the whole cohort and the subgroup subjected to whole blood HNL analysis (Tables S1 and S2).

### 3.2. Human Neutrophil Lipocalin in Plasma and Whole Blood

**Figure 1:**
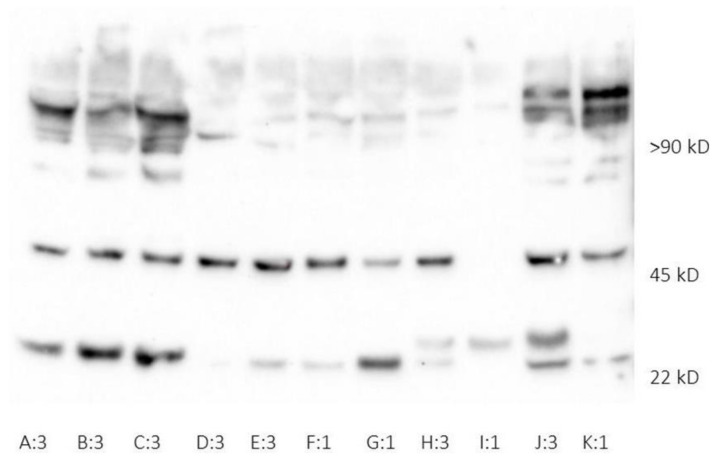
Western blot of plasma samples during the first week of ICU treatment. Monomeric (22 kD), homodimeric (45 kD), and heterodimeric (>90 kD) HNL all present in plasma samples collected at admission (1) and on day three (3) during the first week of intensive care. The letters A–K represent individual patients in the cohort. HNL, human neutrophil lipocalin.

HNL was present in both monomeric and dimeric forms in plasma at ICU admission, reflecting epithelial injury and neutrophil activity (Figure 1). The median HNL concentrations in plasma (P-HNL Dimer) were 9.54 (7.46) and 11.1 (7.08) µg/L at admission and day three, respectively, and the concentrations were not significantly different from one another (*p* = 0.389).

The median concentration of whole blood HNL (B-HNL) at the time of the first sample was 96.6 (71.2) µg/L. Over the first three weeks, there was a significant increase in B-HNL (*p* = 0.016). The median B-HNL measured at peak concentrations was 134 (101) µg/L. Cutoff values were calculated and concentrations close to those for AKI stage 3 were universally implemented, 13.2 µg/L and 190 µg/L for P-HNL Dimer and peak B-HNL, respectively.

### 3.3. Neutrophil Count, NLR, and HNL

The observed peak neutrophil count was 9.2 (6.2), and the median NLR was 8.1 (7.6). The neutrophil count and NLR were significantly higher in patients above 13.2 µg/L P-HNL Dimer when admitted to intensive care (*p* = 0.026 and *p* = 0.042). P-HNL Dimer correlated with neutrophil count and NLR at admission (*r* = 0.387, *p* < 0.001 and *r* = 0.327, *p* = 0.003) (Figure S3).

The peak neutrophil count and peak NLR were significantly higher during ICU stay in patients above 190 µg/L B-HNL (*p* = 0.001 and *p* = 0.004). B-HNL correlated well with peak neutrophil count and NLR (*r* = 0.614, *p* < 0.001 and *r* = 0.514, *p* = 0.000).

Neutrophil count at admission did not differentiate between outcomes. However, the peak neutrophil count was higher in all but one of the outcomes; invasive ventilation (*p* < 0.001), vasopressor treatment (*p* < 0.001), thrombosis (*p* = 0.033), AKI of any stage (*p* = 0.015), AKI stage 3 (*p* = 0.004), and CRRT (*p* < 0.001). The exception was 30-day mortality (*p* = 0.096).

Peak NLR predicted AKI stage 3 (*p* = 0.003), CRRT (*p* = 0.001), invasive ventilation (*p* = 0.009), vasopressor treatment (*p* = 0.003), and 30-day mortality (*p* = 0.023).

### 3.4. HNL and Acute Kidney Injury

**Figure 2:**
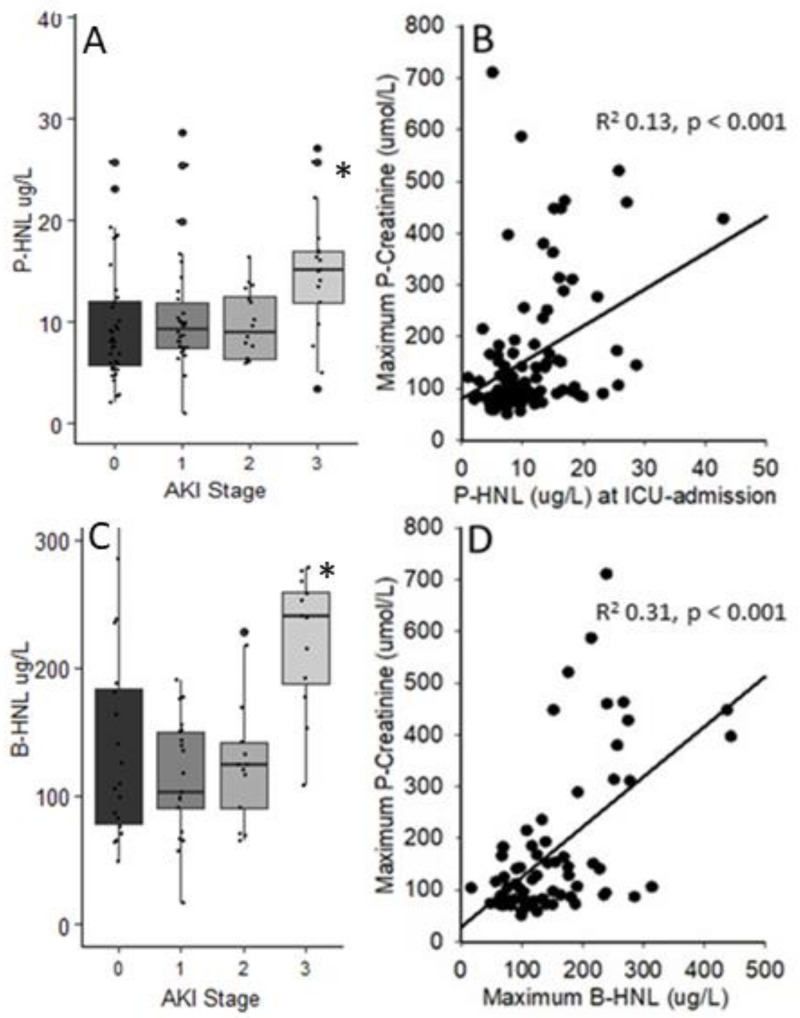
Human neutrophil lipocalin in plasma at admission and peak concentrations in whole blood during intensive care vs. acute kidney injury stage and plasma creatinine. A one-way ANOVA (**A**) demonstrating that P-HNL Dimer at admission differed between AKI stages (*p* = 0.010); however, post-hoc analysis indicated the main difference to be between no AKI and AKI stage 3 * (*p* = 0.006, Bonferroni-adjusted). The plasma creatinine concentration increased as P-HNL Dimer increased significantly (**B**). The biomarker could explain 13% of the variation in creatinine. A one-way ANOVA of AKI stage and B-HNL (**C**) was also significant (*p* < 0.001). Post-hoc analysis revealed a difference between no AKI, AKI stage 1, and AKI stage 2 separately with AKI stage 3 * (*p* = 0.002, *p* < 0.001, and *p* = 0.003, Bonferroni-adjusted). Plasma creatinine increased with peak B-HNL concentrations, (**D**) and the biomarker could explain 31% of the variation in plasma creatinine.

P-HNL Dimer at admission was higher in patients that developed AKI than in those that did not (*p* = 0.046). P-HNL Dimer at admission also differed between AKI stages (*p* = 0.010); however, post-hoc analysis indicated the main difference to be between no AKI and AKI stage 3 (*p* = 0.006) (Figure 2). Similarly, AKI stage 3 was separated from those with no AKI and less severe stages of AKI by dimeric HNL (*p* = 0.001). Patients with CRRT had higher P-HNL Dimer concentrations (*p* = 0.002). ROC estimated the cutoff for predicting AKI to 6.02 µg/L (AUC 0.623, 95% CI 0.507–0.739, *p* = 0.039), with a sensitivity of 91.9% and a specificity of 31.6% (Table S3). For AKI stage 3, the cutoff was 13.4 µg/L (AUC 0.751, 95% CI 0.612–0.889, *p* = 0.001) with a sensitivity of 72.2% and a specificity of 80.4%. The cumulative risk of developing AKI of any stage compared to no AKI during ICU stay was significantly higher in patients above 13.2 µg/L of P-HNL Dimer at admission (*p* = 0.035).

**Figure 3:**
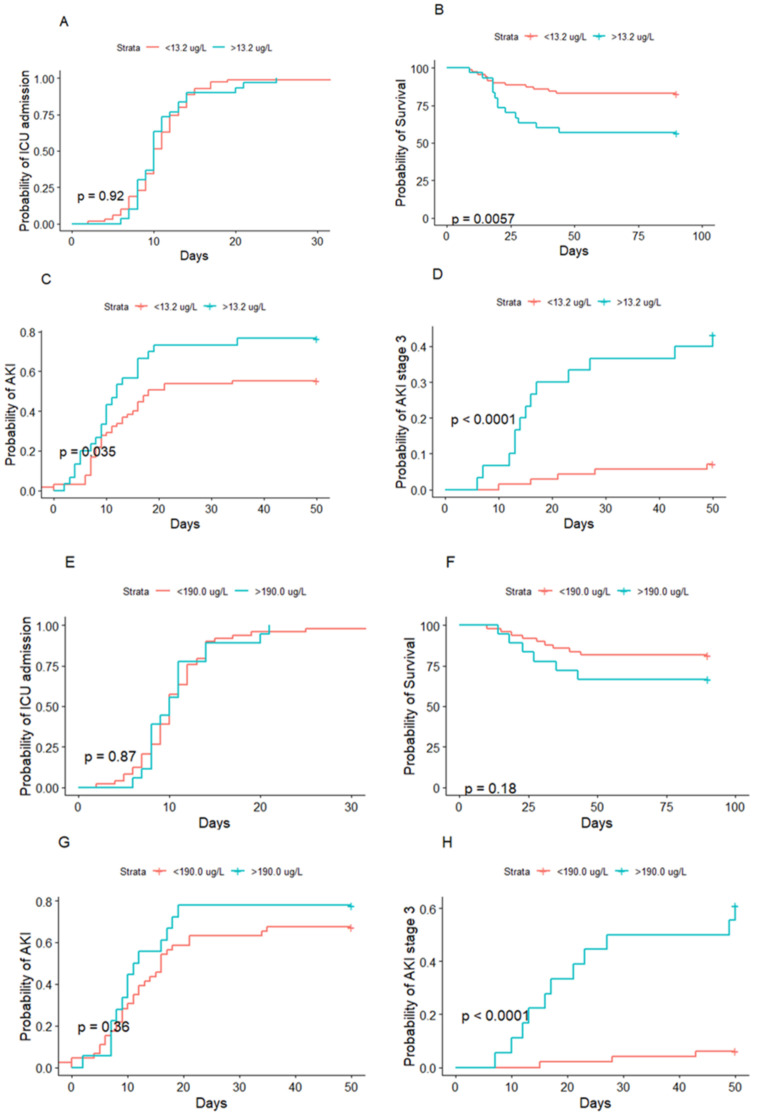
Probability of admission to intensive care, acute kidney injury of any stage, and stage 3 development, as well as mortality vs. human neutrophil lipocalin in plasma at admission (**A**–**D**) and vs. peak concentrations of human neutrophil lipocalin in whole blood (**E**–**H**) during intensive care. ROC in conjunction with Youden’s J statistic were calculated to find suitable cutoff values for HNL in plasma and whole blood. Since the primary focus was severe AKI development, a cutoff close to that calculated from AKI stage 3 was applied to all four outcomes in the figure. The concentrations used were 13.2 µg/L and 190 µg/L for P-HNL Dimer and peak B-HNL, respectively. Kaplan–Meyer curves were than calculated for ICU admission (**A**,**E**), survival (**B**,**F**), AKI (**C**,**G**), and AKI stage 3 (**D**,**H**). Time to ICU admission was not different in the groups above and below the cutoffs for P-HNL Dimer or B-HNL (*p* = 0.92 and *p* = 0.87). The cumulative risk of death, developing AKI of any stage and AKI stage 3 were higher if above 13.2 µg/L in plasma at admission (*p* = 0.0057, *p* = 0.035, and *p* < 0.0001, respectively). The cumulative risk of death or developing AKI did not differ between those above or below 190.0 µg/L B-HNL (*p* = 0.18 and *p* = 0.36, respectively); however, the cumulative risk of developing AKI stage 3 was greater in the patients above the cutoff (*p* < 0.001).

**Figure 4:**
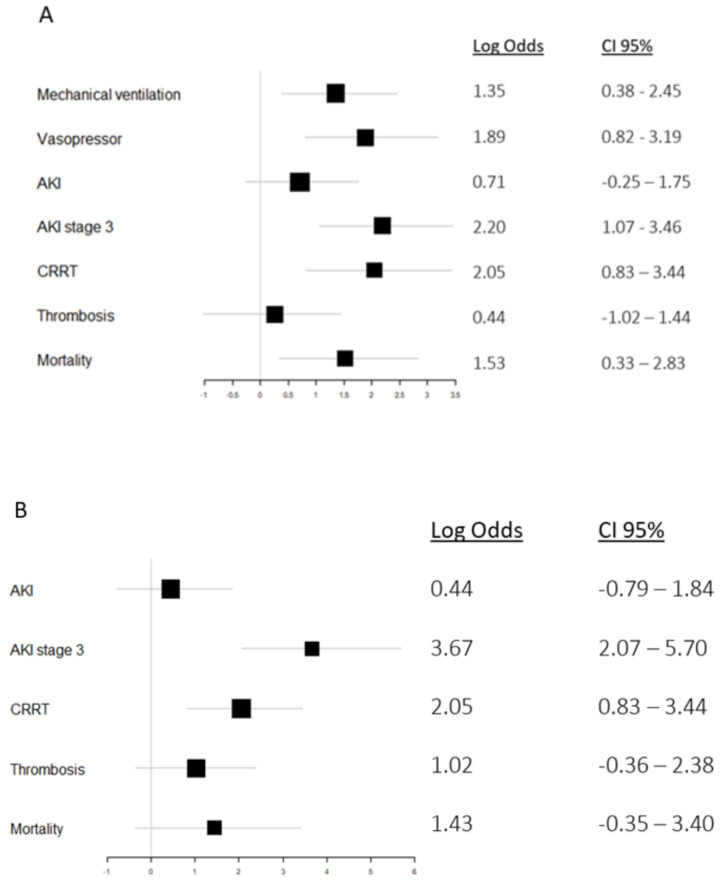
Log odds for outcomes dependent on the concentration of dimeric human neutrophil lipocalin in plasma at admission (**A**) and peak concentration of human neutrophil lipocalin in whole blood (**B**) during intensive care. For each outcome, a logistic regression was performed for P-HNL Dimer at admission and peak B-HNL during intensive care. The biomarker was used as a categorical variable, either above or below the cutoff of 13.2 µg/L and 190 µg/L, respectively, and the regression was adjusted for age. Patients with P-HNL Dimer and peak B-HNL concentrations above said cutoffs demonstrated increased risks of developing AKI stage 3 (8.99 and 39.2 respectively) and renal replacement therapy (7.77 for both).

Likewise, the cumulative risk of developing AKI stage 3 was also distinguished in the patients above the cutoff (*p* < 0.001) (Figure 3). The relative risk of developing AKI and AKI stage 3 if above 13.2 µg/L of P-HNL Dimer at admission was 2.03 (95% CI 0.77–5.78) and 8.99 (95% CI 2.91–31.81) times greater, respectively. The risk of CRRT was 7.77 times those below 13.2 ug/L (95% CI 2.30–31.27) (Figure 4A).

There was no difference in the peak B-HNL concentration between patients with AKI and no AKI (*p* = 0.493). However, the B-HNL concentration differed between stages of AKI (*p* < 0.001). Post-hoc analysis revealed that no AKI and AKI stages 1 and 2 were all separated from AKI stage 3 (*p* = 0.002, *p* < 0.001, and *p* = 0.003) (Figure 2). B-HNL was higher in patients with AKI stage 3 and CRRT (*p* < 0.001 and *p* < 0.001). B-HNL, during the first three weeks, was higher in patients with AKI stage 3 (*p* = 0.005) compared to those with no AKI or lower stages. ROC for AKI did not reach significance (AUC 0.564, 95% CI 0.409–0.719, *p* = 0.411).

A concentration of 191 µg/L (AUC 0.894, 95% CI 0.804–0.983, *p* < 0.001) with a sensitivity of 78.6% and a specificity of 88.7% was estimated for AKI stage 3. The cumulative risk of developing AKI at any stage during ICU stay did not differ between those below and above 190 µg/L B-HNL (*p* = 0.36); however, the cumulative risk of developing AKI stage 3 was greater in the patients above the cutoff (*p* < 0.001) (Figure 3). The relative risk of developing AKI was 1.26 (95% CI 0.35–5.90), while the relative risk of AKI stage 3 or CRRT was 39.2 (95% CI 7.92–298.89) or 7.77 (95% CI 2.30–31.27) for B-HNL above 190 µg/L (Figure 4B).

### 3.5. HNL and Mortality

P-HNL Dimer differed between survivors and non-survivors at 30 days (*p* = 0.034) (Figure 3). ROC analysis estimated a cutoff concentration for 30-day mortality of 14.0 µg/L (AUC 0.679, 95% CI 0.530–0.828, *p* = 0.015) with a sensitivity of 57.9% and a specificity of 81.5%. The cumulative risk of death was higher if P-HNL Dimer was above 13.2 µg/L at admission (*p* = 0.0057). The relative risk of 30-day mortality was 4.60 (95% CI 1.39–16.91). B-HNL did not predict 30-day mortality (*p* = 0.087).

### 3.6. HNL, Organ Support, and Thrombosis

**Figure 5:**
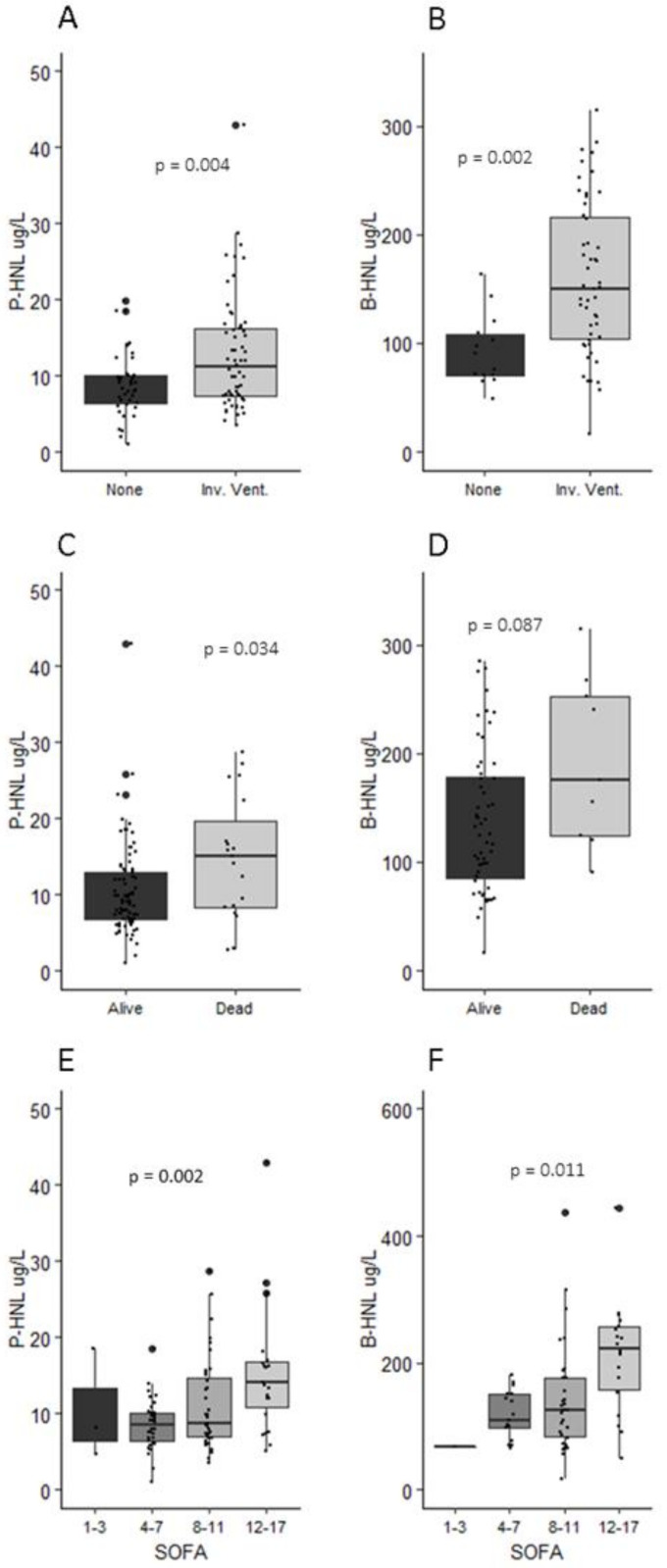
Human neutrophil lipocalin in plasma at admission and peak concentrations in whole blood during intensive care vs. invasive ventilation (**A**,**B**), 30-day mortality (**C**,**D**), and SOFA score (**E**,**F**). P-HNL Dimer at admission differentiated between survivors and non-survivors at 30 days, but peak B-HNL concentration did not (*p* = 0.034 and *p* = 0.087). Those that were treated with invasive ventilation demonstrated higher mean concentrations of P-HNL Dimer and B-HNL (*p* = 0.004 and *p* = 0.002). P-HNL Dimer and B-HNL could both separate between patients with high and low SOFA scores with increasing HNL concentrations associated with higher SOFA scores during ICU stay (*p* = 0.002 and *p* = 0.011).

Patients treated with invasive ventilation or vasopressors had higher P-HNL Dimer levels at admission (*p* = 0.004 and *p* < 0.001) (Figure 3). P-HNL Dimer did not correlate with PaO_2_/FiO_2_. ROC estimated an optimal P-HNL Dimer cutoff for invasive ventilation of 10.3 µg/L (AUC 0.637, 95% CI 0.529–0.746, *p* = 0.020) with a sensitivity of 53.0% and a specificity of 77.5%. The relative risk of invasive ventilation and vasopressors was 3.87 (95% CI 1.46–11.64) and 6.61 (95% CI 2.27–24.33), respectively. P-HNL Dimer did not predict thromboembolism (*p* = 0.833), but could predict high and low SOFA (*p* = 0.002) (Figure 5).

B-HNL peak concentrations predicted both invasive ventilation and vasopressor treatment (*p* = 0.002 and *p* = 0.004). Furthermore, B-HNL above 190 µg/L predicted lower PaO_2_/FiO_2_ (*p* = 0.011). ROC for B-HNL estimated a cutoff of 122 µg/L (AUC 0.799, 95% CI 0.688–0.91, *p* = 0.001) with a sensitivity of 67.9% and a specificity of 85.7%. B-HNL could not differentiate between patients with or without thromboembolism (*p* = 0.161). Increasing B-HNL was associated with higher SOFA (*p* = 0.011) (Figure 5).

## 4. Discussion

The present study demonstrated that persistently elevated neutrophil lipocalin in peripheral plasma and blood, interpreted as increased systemic neutrophil activity, is associated with severe AKI in critical COVID-19. Two thirds of the patients developed AKI during intensive care. P-HNL Dimer at admission and peak B-HNL concentration could clearly distinguish patients with severe AKI and CRRT. A reason for the stronger association of the marker with AKI stage 3 may be that milder forms of AKI in these patients are related to other mechanisms than neutrophil-mediated injury. As is the case with AKI in general, renal dysfunction in COVID-19 is probably multifactorial. It may be mediated by several unrelated mechanisms, including direct viral effects, inflammation, and coagulopathy, and in the intensive care setting, side effects of invasive ventilation and various drugs [3,29]. We previously showed that mild AKI with low urine volume is common in COVID-19-related AKI, associated with hypovolemia, and does not lead to persistent kidney injury [4,30]. Neutrophil-mediated renal injury is not to be expected in these milder forms. However, severe AKI may be, in part, neutrophil-mediated in these patients.

Our results are consistent with previous findings that NLR can predict severe AKI [31]. Both neutrophil count and NLR at peak levels in our cohort strongly differentiated between patients with and without AKI stage 3. The association of HNL, a likely signal of neutrophil priming and activity, with severe AKI is important, since AKI stage 3 is closely associated with adverse outcomes such as mortality and progression of chronic kidney disease [29,32]. We speculate that SARS-CoV-2 initially triggers a local inflammatory process at the primary site of infection, the lungs, and as the disease progresses beyond respiratory dysfunction, this response disseminates to other organs mediated by, but not limited to, neutrophils. As described in lethal COVID-19, neutrophil plugs, aggregates of neutrophils or platelets with NETs are found post-mortem in the kidneys, as well as the heart, liver, spleen, and brain [33]. It may, however, not only be a dysregulated inflammatory response with a spillover effect, but also a direct viral effect due to an extra-pulmonary spread of SARS-CoV-2. Viremia and extra-pulmonary organ infection with subsequent inflammatory responses in these organs could thus be another explanation for increased systemic neutrophil activity. As previously demonstrated, viral plasma load is associated with critical disease and neutrophil activation [34,35]. In the previously mentioned post-mortem study, SARS-CoV-2 was found in organs distant from the lungs. However, AKI in COVID-19 is not clearly associated with renal infection, as indicated by the rare presence of renal SARS-CoV-2 in urine [36]. Activation of peripheral neutrophils is perhaps secondary to viral spread in the body, or a disseminated inflammatory response induced by the pneumonia itself without viremia, both would appear detrimental to the patient.

The majority of patients in our cohort were admitted due to respiratory failure and treated with invasive ventilation, thus fulfilling the Berlin criteria of acute respiratory distress syndrome (ARDS). Neutrophil infiltration of lung tissue is a known contributor to ARDS development, which is consistent with our finding that both HNL in plasma and blood are associated with a greater risk of invasive ventilation [37]. HNL measured in whole blood, indicative of neutrophil priming, was also associated with lower PaO_2_/FiO_2_, further strengthening this association.

Multiple organ failure, as compared to single organ failure in COVID-19, has a worse prognosis [38]. AKI, as a representation of extra-pulmonary organ dysfunction, could be indicative of an incipient multiple organ dysfunction syndrome. This argument is strengthened by the significant relationship between increasing levels of HNL and increasing SOFA scores. AKI has previously been established as an independent risk factor of death in COVID-19 [3]. This is consistent with our finding that P-HNL Dimer at admission predicts mortality. Interestingly, HNL measured in whole blood was not associated with mortality in our data. The reason for this finding is not clear, but may be related to a relatively low number of events in the current investigation.

It has been demonstrated that neutrophils from COVID-19 patients are more prone to release NETs and ROS indicative of increased priming [15,39,40]. During the first week of intensive care, HNL was present both in monomeric (24 kDa) and homodimeric (45 kDa) forms. A likely source of monomeric HNL at this time would be pulmonary epithelium due to lung injury, in addition to neutrophil-released HNL. Increased levels of plasma HNL, not differentiated between monomeric and dimeric forms, have been demonstrated to be associated with AKI severity, CRRT, and mortality in earlier studies [18,41]. The relationship observed in this study between HNL and severe AKI, with an assay aimed at neutrophil-specific dimeric HNL, suggests that neutrophils play a part in the phenomenon of lung–kidney crosstalk in critical care, where the failing of one of the two is often associated with the failing of the other, as has been proposed previously [42,43]. Earlier studies have investigated the use of whole blood and plasma NGAL as biomarkers of AKI development in intensive care, often without discriminating between the different isoforms of the biomarker [44,45]. In the present study, dimeric HNL in plasma early on during intensive care was associated with AKI and CRRT, suggesting that this particular molecular form may have potential as a biomarker of severe AKI.

The main strength of this study is the prospective design that made it possible to analyze HNL in fresh whole blood and the inclusion of the majority of eligible consecutive patients. However, our study has limitations. The immunoassay of neutrophil lipocalin in plasma aimed to detect dimeric HNL; this form, having previously been established as of neutrophil origin, cannot entirely exclude an additional source of dimeric HNL. The whole blood analysis detected both monomeric and dimeric variants of HNL, and hence could reflect neutrophil activity and epithelial injury. The analysis, however, was performed after neutrophil activation and no other active source of HNL was known to be present in sampled blood. The majority of the HNL present in the samples would therefore likely be neutrophil in origin. The single-center design is a limitation that may reduce the applicability of the results in other settings, making validation in other centers a priority.

Although the focus on a cohesive patient group with a single disease in intensive care is a strength for interpreting the role of HNL and neutrophil activity in COVID-19, it precludes conclusions about other patient groups.

In conclusion, the present study suggests that neutrophils are an important mediator of severe AKI in critically ill COVID-19 patients. This is supported by increased plasma levels of dimeric HNL, indicative of neutrophil activity, at admission being associated with an increased risk of AKI, AKI stage 3, and CRRT, in addition to mortality in critical ill patients with respiratory failure due to SARS-CoV-2 infection. I n addition, persistently elevated levels of HNL in peripheral whole blood, interpreted as increased neutrophil priming, was associated with severe AKI and CRRT.

## Figures and Tables

**Table 1:** Patient demographics.

	No AKI (*n* = 36)	AKI Stage 1 and 2 (*n* = 49)	AKI Stage 3 (*n* = 18)	All (*n* = 103)
Age, mean (SD)	55.9 (15.8)	59.3 (14.4)	67.0 (9.0)	60.5 (14.1)
Female, *n* (%)	11 (31%)	15 (30%)	1 (6%)	27 (26%)
BMI, mean (SD) *n* = 89	27.6 (4.5) (*n* = 28)	29.5 (6.0) (*n* = 46)	32.2 (11.0) (*n* = 15)	29.6 (6.0)
Hypertension, *n* (%)	15 (42%)	25 (51%) (*n* = 48)	13 (72%)	53 (52%)
Diabetes mellitus, *n* (%)	7 (19%)	17 (35%) (*n* = 48)	5 (28%)	29 (28%)
Pulmonary disease *n* (%)	11 (31%)	10 (20%) (*n* = 48)	4 (22%)	25 (24%)
Days since symptom onset, median (IQR) *n* = 103	11 (3)	10 (4)	10 (5)	10 (4)
SAPS3, median (IQR) *n* = 101	51 (7) (*n* = 34)	53 (17)	55 (9)	54 (11)
SOFA admission, median (IQR) *n* = 100	4 (3) (*n* = 24)	6 (4) (*n* = 47)	6 (5) (*n* = 15)	5 (4)
AKI, any stage, *n* (%)	na	na	na	67 (65%)
Stage 1, *n* (%)	na	na	na	33 (32%)
Stage 2, *n* (%)	na	na	na	16 (16%)
Stage 3, *n* (%)	na	na	na	18 (17%)
PaO_2_/FiO_2_ ratio at admission, median (IQR) *n* = 99	93.8 (42.8) (*n* = 34)	80.3 (29.3) (*n* = 47)	75.0 (25.5)	80.3 (29.3)
Free days				
ICU, mean (SD)	18.2 (9.5)	13.1 (10.7)	4.2 (7.2)	13.4 (10.8)
Invasive ventilation, mean (SD)	23.0 (10.4)	17.3 (12.9)	7.8 (10.1)	17.6 (12.6)
Vasopressor, mean (SD)	24.3 (10.3)	18.4 (12.7)	9.7 (10.6)	18.9 (12.5)
CRRT, mean (SD)	26.7 (9.6)	20.8 (14.0)	9.6 (11.9)	20.9 (13.5)
30-day mortality, *n* (%)	4 (11%)	10 (20%)	6 (33%)	20 (19.4%)

AKI = Acute Kidney Injury, BMI = Body Mass Index, SAPS3 = Simplified Assessment Physiology Score 3, SOFA = Sequential Organ Failure Assessment, ICU = Intensive Care Unit, CRRT = Continuous Renal Replacement Therapy.

**Table 2:** Routine chemistry and inflammatory markers.

	At Admission	Peak	Reference Values
CRP (mg/L), median (IQR)	168 (109) *n* = 99	286 (191) *n* = 101	<5
Leukocytes (10^9^/L), median (IQR)	7.4 (3.9) *n* = 100	13.4 (8.7) *n* = 101	3.5–9.0
Neutrophils (10^9^/L), median (IQR)	6.0 (4.1) *n* = 84	9.2 (6.2) *n* = 93	1.3–5.4
Procalcitonin (ug/L), median (IQR)	0.46 (0.93) *n* = 93	1.50 (4.14) *n* = 100	<0.05
Ferritin (ug/L), median (IQR)	1284 (2051) *n* = 83	2429 (2635) *n* = 101	25–310
Interleukin-6 (pg/mL), median (IQR)	30.0 (48.7) *n* = 101	na	na
Interleukin-8 (pg/mL), median (IQR)	15.1 (14.1) *n* = 101	na	na
B-HNL (µg/L), median (IQR)	96.6 (71.2) * *n* = 67	134 (101) *n* = 67	95.5 (55)
P-HNL (µg/L), median (IQR)	9.5 (7.5) *n* = 100	11.1 (8.2) *n* = 103	3.6 (2)
NLR, median (IQR)	6.7 (4.9) *n* = 83	8.1 (7.6) *n* = 90	na

CRP = C-reactive Protein, HNL= Human Neutrophil Lipocailin, B = whole blood, P = plasma, NLR = Neutrophil Lymphocyte Ratio. * First sample after ICU admission.

## Data Availability

Individual level data are available from the authors on reasonable request, as detailed at https://doi.org/10.17044/scilifelab.14229410.v1 (accessed on 18 March 2021).

## Supplementary Materials

The following are available online at https://www.mdpi.com/article/10.3390/jcm10184144/s1, Figure S1: Study enrollment flowchart, Figure S2: Correlation of outcomes and concentration of human neutrophil lipocalin in plasma and whole blood during intensive care, Figure S3: Correlations of routine biomarkers and cytokines in plasma with concentration of human neutrophil lipocalin in plasma and whole blood during intensive care, Table S1: Patient demographics in subgroup of patients where human neutrophil lipocalin was analyzed in whole blood, Table S2: Routine chemistry and inflammatory markers in subgroup of patients with human neutrophil lipocalin analysis performed in whole blood, Table S3: Cut off estimates for human neutrophil lipocalin in plasma and blood according to area under the Reciever Operating Curves.
