# Supplementary material for: Systemic Human Neutrophil Lipocalin Associates with Severe Acute Kidney Injury in SARS-CoV-2 Pneumonia
Source: J Clin Med. 2021 Sep 14;10(18):4144. doi: 10.3390/jcm10184144 (PMC8464787; doi:10.3390/jcm10184144)
Supplement: Supplementary file 1 [file jcm-10-04144-s001.zip › jcm-1368940-supplementary.pdf]

Supplementary Materials:

Supplementary Figures

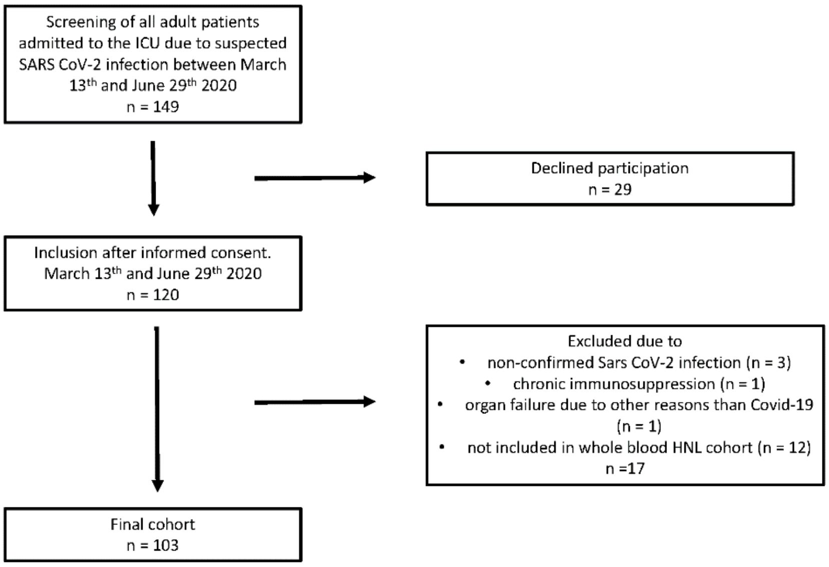

Figure S1. Study enrollment flowchart.

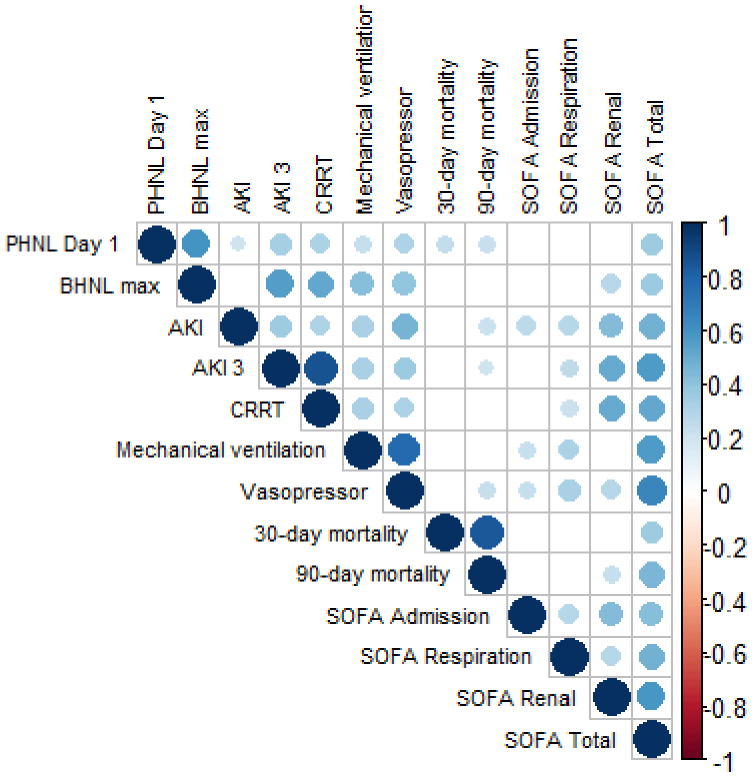

Figure S2. Correlation of outcomes and concentration of human neutrophil lipocalin in plasma and whole blood during intensive care. P-HNL Dimer at admission and peak B-HNL concentration and their correlations with outcomes were calculated with Spearman rank to not violate the assumption of normality. Only positive correlations were found. P-HNL Dimer and B-HNL correlated well with one another (r=0.598, p <0.001). In regards to outcome peak B-HNL demonstrated stronger associations with invasive ventilation (r=0.421, p<0.001), AKI stage3 (r=0.554, p<0.001) and CRRT (r=0.510, p<0.001) than P-HNL Dimer (r=0.233, p=0.020; r=0.334, p=0.001; r=0.315, p=0.001).

The correlation with AKI development was modest for P-HNL Dimer (r=0.215, p=0.031) and not significant for B-HNL (p=0.337). Thirty-day mortality and 90-day mortality had significant correlations with P-HNL Dimer (r=0.243, p= 0.015; r=0.227, p=0.023) but not with peak B-HNL perhaps due to few events. In regards to total SOFA score, both P-HNL Dimer and peak B-HNL reached significant correlations (r=0.350, p<0.001; r=0.366, p=0.002).

(HNL = Human Neutrophil Lipocalin, AKI = Acute Kidney Injury, CRRT = Continuous Renal Replacement Therapy)

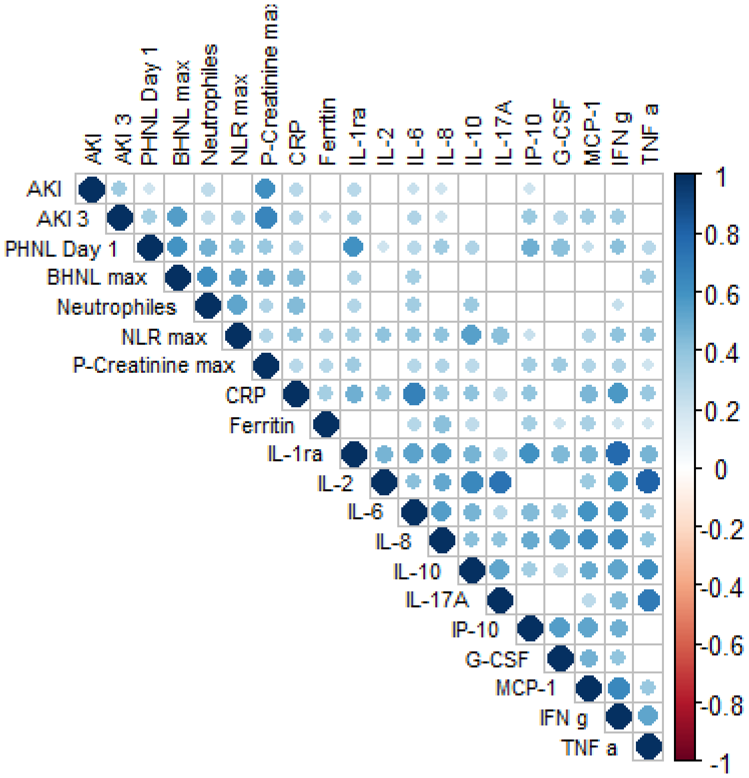

Figure S3. Correlations of routine biomarkers and cytokines in plasma with concentration of human neutrophil lipocalin in plasma and whole blood during intensive care. P-HNL Dimer at admission and peak B-HNL concentration and their correlations with biomarkers were calculated with Spearman rank to not violate the assumption of normality. Only positive correlations were found. Peak neutrophil count during ICU stay (r=0.614, p<0.001; 0.488, p<0.001) and peak NLR correlated stronger with peak B-HNL than P-HNL Dimer (r=0.514, p<0.001; r=0.387, p<0.001). CRP at its peak during ICU stay correlated well with peak B-HNL in comparison to P-HNL Dimer at admission (r=0.436, p<0.001 and r=0.275, p=0.006 respectively). None of them were associated with ferritin. Correlations between cytokines in plasma and P-HNL Dimer at admission were significant but modest in general. The strongest correlations in addition to IL-6 were found with IL-1ra (r=0.600, p<0.001), IL-8 (r=0.329, p=0.001), IL-10 (r=0.306, p=0.007), IP10 (r=0.484, p<0.001), G-CSF (r=0.417, p<0.001) and IFN γ (r=0.413, p<0.001). However, the cytokine profile at admission generally correlated better with P-HNL Dimer than peak B-HNL perhaps an effect of timing since the latter often was observed later on during intensive care. Peak B-HNL only correlated with two cytokines in addition to IL-6; L-1ra (r=0.314, p=0.012) and TNF α (r=0.350, p=0.005). IL-6 had a significant but modest relationship with both P-HNL Dimer and B-HNL (r=268, p=0.008; r=0.332, p=0.007). The peak neutrophil count correlated with IL-1ra (0.309, p=0.003), IL-6 (r=0.367, p<0.001), IL-10 (r=0.389, p=0.001), and IFN γ (r=0.256, p=0.015). NLR at peak neutrophil concentration demonstrated the most numerous cytokine correlations; IL-1ra (r=0.330, p=0.002), IL-2 (r=0.404, p<0.001), IL-6 (r=0.399, p<0.001), IL -8 (r=0.393, p<0.001), IL-10 (r=0.546, p<0.001), IL-17A (r=0.418, p<0.001), IP10 (r=0.227, p=0.035), MCP1 (r=0.293, p=0.006), IFN γ (r=0.404, p<0.001) and TNF α (r=0.410, p<0.001).

Supplementary Tables

Table S1. Patient demographics in subgroup of patients where human neutrophil lipocalin was analyzed in whole blood.

Age, mean (SD)	59.6 (13.5)
Female, n(%)	17 (25%)
BMI, mean (SD)	29.4 (6.1)
Hypertension, n(%)	32 (48%)
Diabetes mellitus, n(%)	17 (25%)
Pulmonary disease n(%)	17 (25%)
Days since symptom onset, median (IQR)	10 (4)
SAPS3, median (IQR)	52 (11)
SOFA admission, median (IQR) n=59	5(3)
AKI, any stage, n(%)	48 (72%)
Stage 1, n(%)	21 (31%)
Stage 2, n(%)	13 (19%)
Stage 3, n(%)	14 (21%)
PaO_2/FiO_2 ratio at admission, median (IQR) n=66	77.3 (27.0)
Free days	
ICU, mean (SD)	11.3 (9.8)
Invasive ventilation, mean (SD)	16.2(11.8)
Vasopressor, mean (SD)	18.2 (11.8)
CRRT, mean (SD)	21.0 (13.3)
30-day mortality, n(%)	9 (13%)

BMI = Body Mass Index, SAPS3 = Simplified Assessment Physiology Score 3, SOFA = Sequential Organ Failure Assessment, ICU = Intensive Care Unit, CRRT = Continuous Renal Replacement Therapy.

Table S2. Routine chemistry and inflammatory markers in subgroup of patients with human neutrophil lipocalin analysis performed in whole blood.

	At admission	Peak	Reference values
CRP (mg/L), median (IQR)	171 (118) n=63	319 (172) n=65	<5
Leukocytes (10^9/L), median (IQR)	7.8 (3.7) n=64	14.3 (12.0) n=66	3.5-9.0
Neutrophils (10^9/L), median (IQR)	6.0 (3.7) n=50	9.8 (7.2) n=58	1.3-5.4
Procalcitonin (ug/L), median (IQR)	0.46 (0.93) n=59	1.70(4.45) n=65	<0.05
Ferritin (ug/L), median (IQR)	1647 (1890) n=56	2488 (2288) n=66	25-310
Interleukin-6 (pg/mL), median (IQR)	33.5 (54.6) n=66	na	na
Interleukin-8 (pg/mL), median (IQR)	16.3 (13.7) n=66	na	na
B-HNL (µg/L), median (IQR) Sample 1*	96.6 (71.2)* n=67	134 (101) n=67	95.5 (55)
P-HNL (µg/L), median (IQR) Sample 1*	9.64 (8.69)* n=66	11.9 (8.35) n=67	3.6 (2)
NLR, median (IQR)	6.4 (4.7) n=50	8.0 (7.6) n=56	na

CRP = C-reactive Protein, HNL= Human Neutrophil Lipocailin, B = whole blood, P = plasma, NLR = Neutrophil Lymphocyte Ratio.

Table S3. Cut off estimates for human neutrophil lipocalin in plasma and blood according to area under the Reciever Operating Curves.

	Outcome	AUC (CI 95%, p)	Estimated cut off	Sensitivity	Specificity
P-HNL at admission	AKI	0.623 (0.507-0.739, p=0.039)	6.02	0.92	0.32
	AKI Stage 3	0.751 (0.612-0.889, p=0.001)	13.4	0.72	0.80
	Invasive ventilation	0.637 (0.529-0.746, p=0.020)	10.3	0.53	0.78
	30-day mortality	0.679 (0.530-0.828, p=0.015)	14.0	0.58	0.81
	90-day mortality	0.651 (0.520-0.782, p=0.024)	11.9	0.60	0.72
	CRRT	0.762 (0.625-0.900, p=0.070)	13.4	0.71	0.78
B-HNL peak	AKI	0.564 (0.409-0.719, p=0.411)	88.5	0.83	0.35
	AKI Stage 3	0.894 (0.804-0.983, p<0.001)	191.4	0.79	0.89
	Invasive Ventilation	0.799 (0.688-0.910, p=0.001)	121.7	0.68	0.86
	30-day mortality	0.693 (0.524-0.863, p=0.063)	118.2	0.89	0.47
	90-day mortality	0.635 (0.469-0.800, p=0.114)	239.5	0.40	0.92
	CRRT	0.898 (0.821-0.975, p<0.001)	151.7	1.00	0.71

HNL = Human Neutrophil Lipocalin, P = plasma, B = blood, AKI = Acute kidney injury, CRRT = Continuous Renal Replacement Therapy.
